# Catalysis of the Oxygen Evolution Reaction by 4–10 nm Cobalt Nanoparticles

**DOI:** 10.1007/s11244-018-0923-4

**Published:** 2018-04-09

**Authors:** Edward Locke, Shan Jiang, Simon K. Beaumont

**Affiliations:** Department of Chemistry, University of Durham, South Road, Durham, DH1 3LE UK

**Keywords:** Cobalt, Nanoparticles, Water splitting, Oxygen evolution reaction, Particle size

## Abstract

Electrolysis of water is key technology, not only for clean energy production, but to ensure a continued supply of hydrogen beyond fossil resources, essential to the manufacture of many chemical goods other than fuels. Cobalt nanomaterials have been widely identified as a promising candidate for the anode (oxygen evolution) reaction in this process, but much work has focused on applied materials or electrode design. Given the importance of oxidation state changes Co(III) → Co(IV) in the accepted reaction mechanism, in this work we look at size effects in small (4–10 nm) cobalt nanoparticles, where the ease of oxidation for lower cobalt oxidation states is known to change with particle size. To discriminate between geometric and chemical effects we have compared the catalysts in this study to others in the literature by turnover frequency (widely used in other areas of catalysis), in addition to the more commonly employed performance metric of the overpotential required to produce a current density of 10 mA cm^−2^. Comparisons are drawn to key examples of using well defined nanomaterials (where the surface are of cobalt sites can be estimated). This has enabled an estimated intrinsic turnover rate of ~ 1 O_2_ molecule per surface Co atom per second at an overpotential of 500 mV in the oxygen evolution reaction under typical alkaline reaction conditions (pH 14.0) to be identified.

**Electronic supplementary material:**

The online version of this article (10.1007/s11244-018-0923-4) contains supplementary material, which is available to authorized users.

## Introduction

Hydrogen, a molecule essential for the production of many major chemicals, is currently produced almost exclusively from methane. However, in the face of concerns about diminishing fossil fuel reserves and energy security, considerable effort has been made to identify renewable sources of hydrogen, and in particular in the area of electrochemical and photochemical water splitting [1, 2]. Electrochemical water splitting would provide a means to harness renewable electricity (wind, tidal, photovoltaic) to produce hydrogen, whereas photochemical water splitting would perform both capture of solar energy and subsequent water splitting [3]. In water splitting technology, the production of oxygen at the anode, known as the oxygen evolution reaction (OER), is generally the more energy demanding/inefficient step [4]. An effective catalyst to reduce the activation energy or overpotential (η) is therefore required. A great deal of work has been conducted to identify materials for this purpose, and while precious metal oxides such as iridium or ruthenium are effective catalysts, there has been a push to identify lower-cost, earth-abundant catalysts for widespread use [4]. Work on thin films of metal oxide catalyst materials based on first row transitions metal oxides found activity Ni > Co > Fe > Mn to follow the reverse order of M–OH bond strength [5, 6], but a plethora of reports have appeared based on low overpotentials for different alloyed first row transition metals, generally working under alkaline conditions [7, 8]. Of particular note for producing stable, active catalysts are cobalt oxides and spinels [9–12], in a number of cases in combination with auxiliaries such as phosphate ions [13–15]. While not generally able to operate under the less alkaline or acidic conditions seen for Ir and Ru based materials because the transition metal oxides dissolve [16], cobalt may represent an acceptable compromise of cost and performance (prices per kg in May 2017: Co: $55, Ru $2090, Ir $29,100) [17]. The phosphate system, known as “Co–Pi” is of interest as electrolysis of Co^2+^ salts in phosphate buffer has been able to produce a single system active for both hydrogen evolution reaction (HER) and OER at lower pHs [14, 18]. Here it has been postulated that, for OER, the phosphate acts as a proton acceptor, favoring the Co(IV) oxidation state thought to be the active species in other cobalt OER catalysts [19]. More generally, a range of cobalt nanomaterials (usually under alkaline conditions, pH 13–14) have been studied, including thin films [6], nanoparticles deposited on Ni foams [20, 21] or carbon materials [9, 22, 23] and mesoporous cobalt oxides [24].

**Fig. 1:**
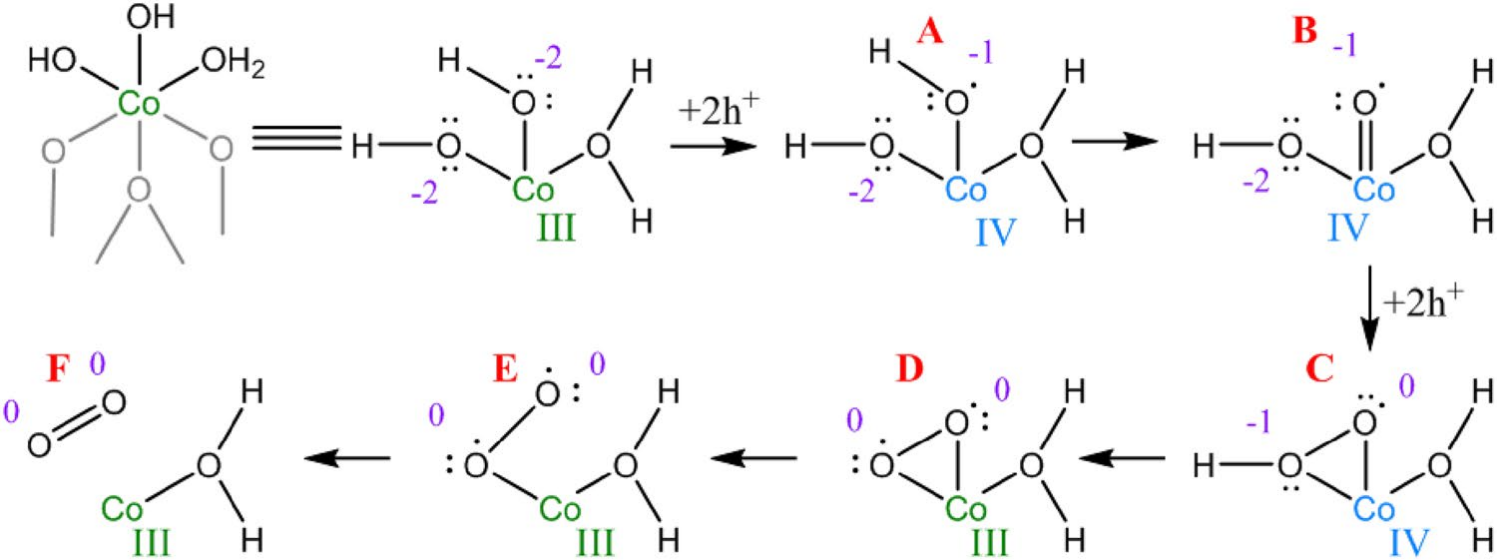
Cobalt catalyzed OER mechanism showing the surface radical intermediates on the injection of holes from the external circuit into incomplete cubane units of cobalt oxide: **a** terminal Co–OH; **b** Co=O oxyl; **c** Co(OOH) hydroperoxo; **d** Co(OO) peroxo; **e** Co–OO superoxo; **f** solvated O_2_ molecule. Cobalt oxidation states are given in Roman numerals and oxidation state of oxygen atoms in Arabic numerals by each species. Reproduced with permission from Mattioli et al. [25]

The above idea that Co(IV) is the key active component has been supported by density functional theory (DFT) calculations, showing Co(IV)-oxyl moieties to be key precursors to the oxygen–oxygen bond forming mechanism in the case of the Co–Pi (also known as CoCat) system. The mechanism for one of the lower energy pathways they identified is shown in Fig. 1, highlighting the changes in cobalt oxidation state at the active site [25]. Strikingly, electron paramagnetic resonance investigations to identify this widely postulated Co(IV) species found only small (< 10%) quantities of Co(IV) in films of the oxide electrocatalysts [26]. This suggests that if the Co(IV) is key for activating the reaction process, then the ease of cobalt oxidation may be of paramount importance in preparing effective catalysts. Nanoparticle size effects and especially particle size dependent redox behavior has been a recent focus of work in the group of Prof. Gabor Somorjai, in honor of whom it is pleasure to contribute a paper to this issue. It is well known from other types of catalysts that small nanoparticles in general may stabilize higher oxidation states of metals [27], or increase their rate of formation [28]. For cobalt specifically, small particles (less than about 6 nm in diameter) are less active in Fischer–Tropsch type chemistry, where larger Co particles to avoid adsorbate induced restructuring or oxidation of the active metallic Co(0) are needed [29–31]. Although particle size effects have been studied for OER, this was upwards from ~ 6 nm [21], so if a similar size threshold exists for the Co(III)/Co(IV) redox couple it would be valuable to extend this range to slightly smaller nanoparticles.

The benchmarking of electrocatalysts for OER using ‘the overpotential required to obtain an electrode current of 10 mA cm^−2^’ has been adopted on a widespread basis, representing the current density required for a 10% solar-to-fuels device [32]. Technologically speaking, this has clear advantages, but in understanding the atomic level chemistry this becomes problematic, as roughness and porosity of the electrode surface mask the intrinsic catalytic properties of the material. A better material could be better either because of improved active site behavior or because of structure supporting a greater number of active sites on the 1 cm^2^ geometric surface area of the electrode for which the current density is measured. Much as turnover frequency (TOF) has served to provide understanding of the chemistry underlying mass activities at a given temperature in conventional thermal catalysis, it can be suggested that their potential in better understanding electrochemical materials’ performance (current density at a given overpotential) is similar, yet only sometimes considered.

In the present work we have therefore studied the OER reaction catalyzed by cobalt nanoparticles in the size range 4–10 nm to identify if any changes in activity exist in this size range, where cobalt oxidation behavior at lower oxidation states is known to vary. Particular attention has been paid to identifying the best strategy to study particle size effects; both a conventional “Tafel plot” based measurements and a more direct surface area normalized method have been used. Furthermore, we have sought to identify an estimate for the intrinsic turnover rate per surface cobalt for these cobalt nanoparticles at a specific overpotential and compared it to that calculated for other recent cobalt nanomaterials in the literature.

## Experimental

Co_2_(CO)_8_ (96% with hexane stabiliser) was obtained from Alfa Aesar, *o*-dichlorobenzene (99%) and Nafion perfluorinated resin solution (5 wt% in alcohol) were obtained from Sigma Aldrich. The *o*-dichlorobenzene was dried by refluxing with CaH_2_ for 3 h and distillation under nitrogen. Oleic acid (97%) was obtained from Acros Organics. All other reagents and solvents were obtained from Fisher Scientific and used without further purification. High purity water was obtained from a Purite Neptune purification system (18.2 MΩ). Synthesis was carried out using standard Schlenk techniques under a nitrogen atmosphere. The inert gas was dried by passing through a moisture trap consisting of indicating phosphorus pentoxide and calcium chloride drying agents. All glassware was washed in a base bath (1 M potassium hydroxide in 2-propanol and water) and then rinsed with water, washed in an acid bath (1 M nitric acid) and again thoroughly rinsed with water before being air dried and then dried in a 100 °C oven overnight before use. Glassware for use in electrochemical experiments was further cleaned as outlined below.

### Nanoparticle Synthesis

Synthesis of 4–10 nm cobalt nanoparticles with narrow size distribution was conducted using a previously reported method [30]. All manipulations with Co_2_(CO)_8_ were carried out in a glove box prior to dissolving in *o*-dichlorobenzene under N_2_. After evacuation of oleic acid (210 mg, 0.743 mmol) for 10 min, anhydrous *o*-dichlorobenzene (20 mL) was added under N_2_. The flask was equipped with a long Liebig condenser and gas release line to accommodate the large volume of carbon monoxide gas which is produced upon decomposition of the carbonyl precursor. While vigorously stirring, the oleic acid solution was heated to the required temperature (as monitored by a submerged K-type thermocouple). Temperature was controlled (± 0.4 °C) using a Omega CN7500 PID controller connected to an isomantle heater. Once stabilized at the required temperature (168, 172, 178 or 183 °C), Co_2_(CO)_8_ in anhydrous *o*-dichlorobenzene (4 mL) was injected quickly into this solution. The solution immediately turned black indicating the formation of colloidal particles. This colloidal suspension was then aged for 20 min prior to stopping heating and cooling the flask in air. *O*-dichlorobenzene (10 mL) and 2-propanol (99.5%, ca. 100 mL) were added to this suspension, precipitating nanoparticles, which could then be extracted by centrifugation (5000 rpm). The solid was re-dispersed and stored in hexane (98.93%) until further use. The inductively coupled plasma optical emission spectroscopy (ICP-OES) derived cobalt yield was found to be 45, 54, 61 and 76% for 3.7, 4.4, 6.3 and 9.3 nm mean diameter nanoparticle samples.

### Electrocatalysis

Electrochemical data were recorded on a Metrohm Autolab PGSTAT128N computer controlled potentiostat using NOVA 1.8 software. All measurements were performed in a one compartment cell using a standard three electrode configuration. It should be noted that care was taken in the placement of the working and counter electrodes to maximise the separation so as to prevent transfer of produced gases to the opposite electrodes. Catalytic runs were collected in rapidly stirred 1 mol dm^−3^ KOH electrolyte (~ 30 mL) at room temperature (20 °C) and referenced to a Ag/AgCl reference electrode (Mettler Toledo InLab). Coiled Pt wire (~ 8 cm long, 0.127 mm diameter, Alfa Aesar) served as the counter electrode. Working electrodes composed of a glassy carbon electrode (BAS instruments MF-2012 with a 3.0 mm working diameter) loaded with cobalt nanoparticles, as detailed below, completed the standard three-electrode configuration. Prior to electrolysis, the solution was saturated with N_2_ via a glass tube for at least 20 min to remove oxygen, enabling direct comparison with prior literature [21]. (Although it has been suggested O_2_ saturation is preferable as it maintains a constant oxygen concentration, even over the stability experiments of 2 h the electrodes produce only a limited number of bubbles compared to the gas flow required to saturate a solution and only minimal amounts are produced during cyclic voltammetry from which the results have been primarily derived). All cyclic voltammograms were obtained with a scan rate of 10 mV s^−1^. Current densities were obtained by dividing by the geometric area of the glassy carbon electrode (0.0707 cm^2^). Chrono-potentiometry was used to assess sample stability by maintaining the current density at the working electrode at 10 mA cm^−1^ showing reasonable stability over several hours for the most active electrode (smallest particle size). Further details of this are discussed in the supporting information along with additional experiments using Nafion stabilizer commonly used for catalysts that do not easily attach to the electrode [33], but which in our hands was found to inhibit activity, but not stabilise the samples significantly.

To thoroughly clean the glassware a weak solution of permanganic acid and manganese heptoxide (commonly known as ‘green acid’) was prepared by slowly adding KMnO_4_ (1–2 crystals) to H_2_SO_4_ (33%, 10 mL). *Note: The concentration should not be increased as manganese heptoxide can decompose explosively*. This was contacted with all internal glass surfaces and left to soak overnight, before washing thoroughly in high purity water to remove all traces of the cleaning agent.

The glassy carbon working electrode was first polished to a mirror like finish using a 0.05 µm alumina polishing disk (Blue Helix) moistened with high purity water. The electrode was then sonicated in high purity water, acetone and high purity water (5 min ea.) and dried in air. The Pt wire counter electrode was cleaned by immersion in nitric acid (70%) for ~ 10 s followed by rinsing in high purity water, sonicating in high purity water and drying in air. The Ag/AgCl reference electrode was cleaned by rinsing thoroughly in high purity water and air drying, and stored in 3 mol dm^−1^ KCl when not in use.

Cobalt nanoparticles were loaded onto the electrode by a simple drop casting method. A sample of the nanoparticle solution in hexane was diluted such that a 50 µL aliquot contained the desired mass of cobalt (based on a solution Co concentration known from ICP-OES). To ensure complete dispersion of the solution, the sample was mixed on a vortex mixer (2 min) and sonicated (10 min) prior to removal of a 50 µL aliquot, which was applied one drop at a time under a bright lamp to evaporate the solvent. Once all the solution had been applied, the electrode was dried at 80 °C for 1 h to cast the nanoparticles to the surface and remove all traces of solvent. Since the electrode is embedded in a plastic holder the mass loading was scaled to account for the fact the solution dries across the whole electrode assembly surface, not just the active electrode area. The experiment in the Electronic Supplementary Material in which a Nafion stabilizer was tested was prepared in a similar manner, but with 5 µL (0.1 wt%) Nafion solution deposited on top of the sample prior to drying. Nafion stabilizer was not used in any of the other experiments described.

For the OER reaction in a pH 14.0 solution and a reference electrode using 3 mol dm^−3^ KCl, E° = 0.404 V versus RHE, then offset 0.210 V to account for the Ag/AgCl reference electrode. Overpotential and current densities were calculated by obtaining a linear plot 10 points either side of the point of interest and using the linear equation of a least squares fit to determine the exact value. TOF per cobalt atom calculations assume 100% faradaic efficiency with 4 electrons per oxygen molecule produced and the amount of cobalt present is derived from ICP-OES analysis. TOF per surface cobalt atom was then obtained by estimating the geometric area, assuming the particles are spherical, of the surface of Co_3_O_4_ nanoparticles (density ratio Co:Co_3_O_4_ is 1.46:1) [34] and using the fact there are 3.1 × 10^18^ cobalt atoms m^−2^ in a typical Co_3_O_4_ surface [based on (100) facets] to obtain an estimate of the number of surface atoms. A sensitivity analysis for the assumptions that particles are spherical and (100) surfaces are exposed is provided in the Electronic Supplementary Material (Table S5), and confirms within the other errors of the experiment the differences obtained are relatively small for cubic or different low energy facets [(111) and (100) that likely dominate the surface]. Co_3_O_4_ is assumed to be present as the stable highest oxidation state form of the nanoparticles before the application of electrical potential. This approach has also been taken for calculation of values based on available literature data where the geometric nanoparticle surface area where possible, or the BET surface area for mesoporous materials, has been used, as tabulated in the supporting information.

### Transmission Electron Microscopy (TEM)

The size of the as prepared nanoparticles was characterized by TEM by casting one droplet of nanoparticle solution (hexane) onto a holey carbon coated copper grid (Agar Scientific) and allowing to evaporate to dryness. TEM imaging was performed using a JEOL 2100F FEG TEM with a Schottky field emission source. The accelerating voltage was 200 kV. The obtained particle size distribution was obtained from imaging at least 6 different areas of the grid and measuring the diameter of over at least 120 individual nanoparticles. ImageJ software was used for manual size analysis of the images. Measurements were taken of the ~ 20 particles in the top right hand corner of each image to ensure consistency and each particle was measured horizontally on the image to avoid systematically measuring the longest axis. No measurable change in particle size was seen in different regions of the grid.

### ICP-OES

ICP-OES was performed on a Jobin Yvon Horiba Ultima-2, with a vertically orientated radial torch and a sequential monochromator. A cobalt in nitric acid ICP-OES standard (Sigma-Aldrich), and the Co emission line at 228.616 nm were used. ICP-OES samples were prepared by dissolving 1.0 mL aliquots of the nanoparticle samples in hexane in 3 mL of aqua regia (HNO_3_:HCl, 1:3 v/v by volume). These samples were then heated to near boiling for ~ 10–20 min to evaporate all the hexane and until it was seen any precipitated metal was seen to be fully dissolved. After cooling, the sample was made up to 25 mL in a volumetric flask using high purity water. The aliquots were calculated such that the concentrations of the 25 mL samples measured should be ~ 80 mg L^−1^, assuming a 60% yield.

## Results and Discussion

**Fig. 2:**
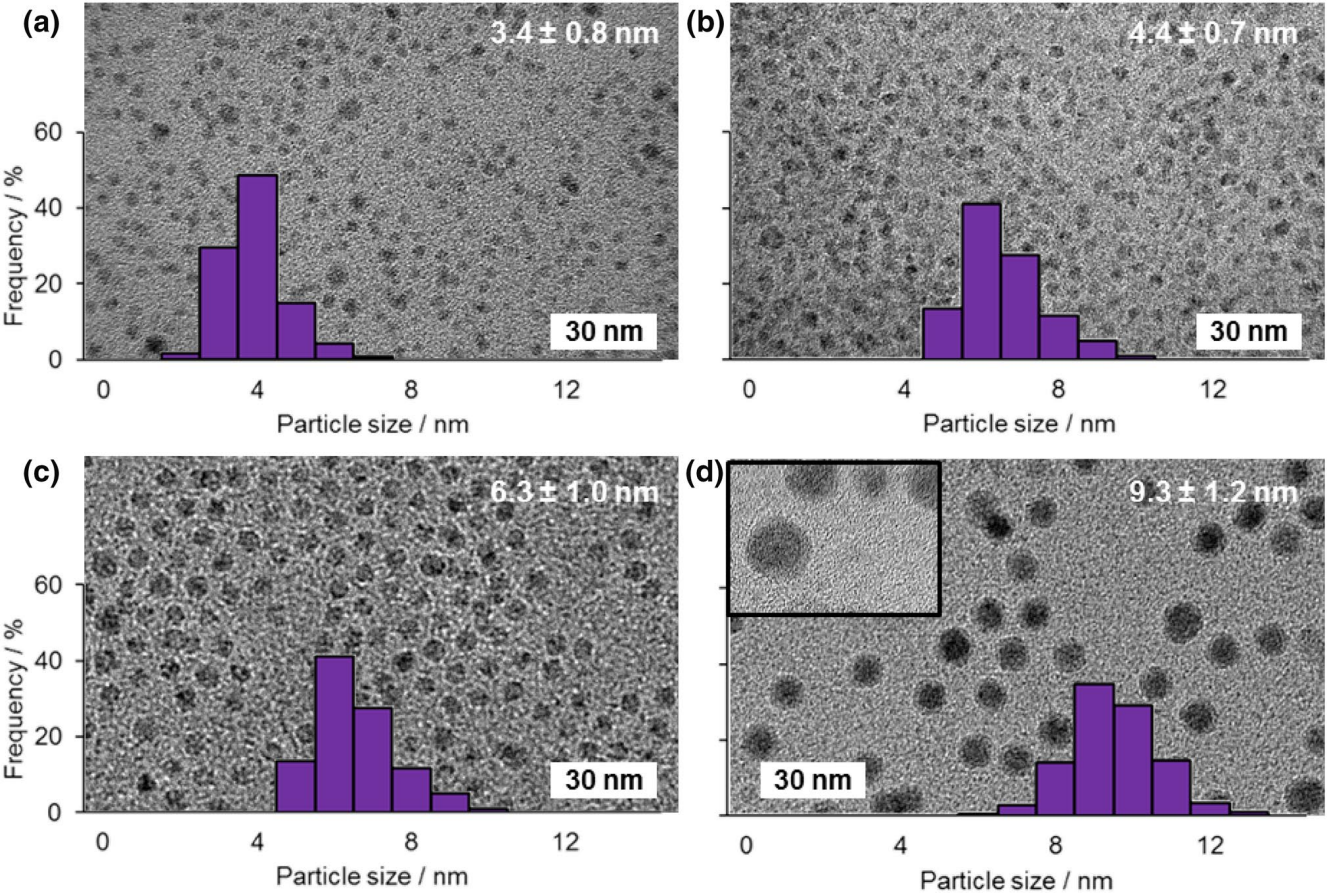
Typical TEM images of each nanoparticle sample overlaid with the particle size distributions obtained from measuring > 120 nanoparticles (**a**–**d** show particles synthesised at 183, 178, 172 and 168 °C producing particles with mean diameters of 3.7 ± 0.8, 4.4 ± 0.7, 6.3 ± 1.0 and 9.3 ± 1.2 nm, respectively). The inset in **d** shows more clearly the oxide layer that forms around the cobalt nanoparticles due to air handling

Representative TEM images of the as synthesized cobalt nanoparticles (Fig. 2) show the formation of a size series (as expected from the literature) of nanoparticles with narrow size distributions as a result of varying the temperature during nanoparticle synthesis. It is notable that, by the time the samples are subjected to electron microscopy, a significant oxide shell forms on the surfaces of the nanoparticles; this can be clearly seen in the case of the largest nanoparticles, as shown in the inset in Fig. 2d. Because of previous findings under similar basic conditions showing there is a negligible difference in activity when starting with Co_3_O_4_, CoO and Co metal nanoparticle coated electrodes, and the ease of surface oxidation seen for nanoparticles of this type, it is expected that such an oxide surface layer will not be important in determining the state of the anode under working conditions once voltage is applied [20].

**Fig. 3:**
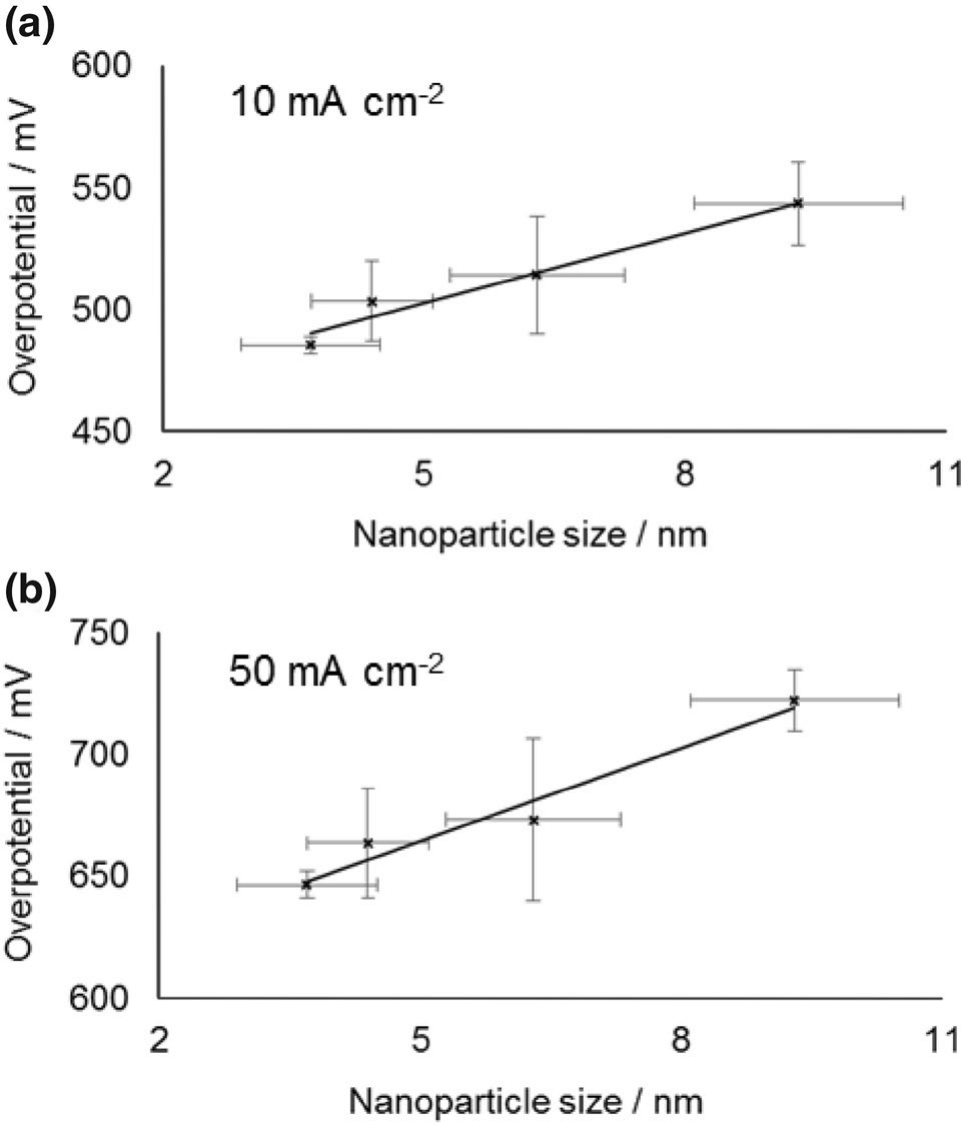
Average overpotential as a function of cobalt nanoparticle size measured at 10 mA cm^−2^ (**a**) and 50 mA cm^−2^ (**b**) using a constant cobalt loading on the electrode of 20 µg cm^−2^. The error bars in the y-axis show standard deviation in the mean based on repeat measurements of each point, the error bars in the x-direction show the standard deviation value from the mean of the measured particle size distribution as described above (and not an error in the mean particle size that would be much smaller)

**Fig. 4:**
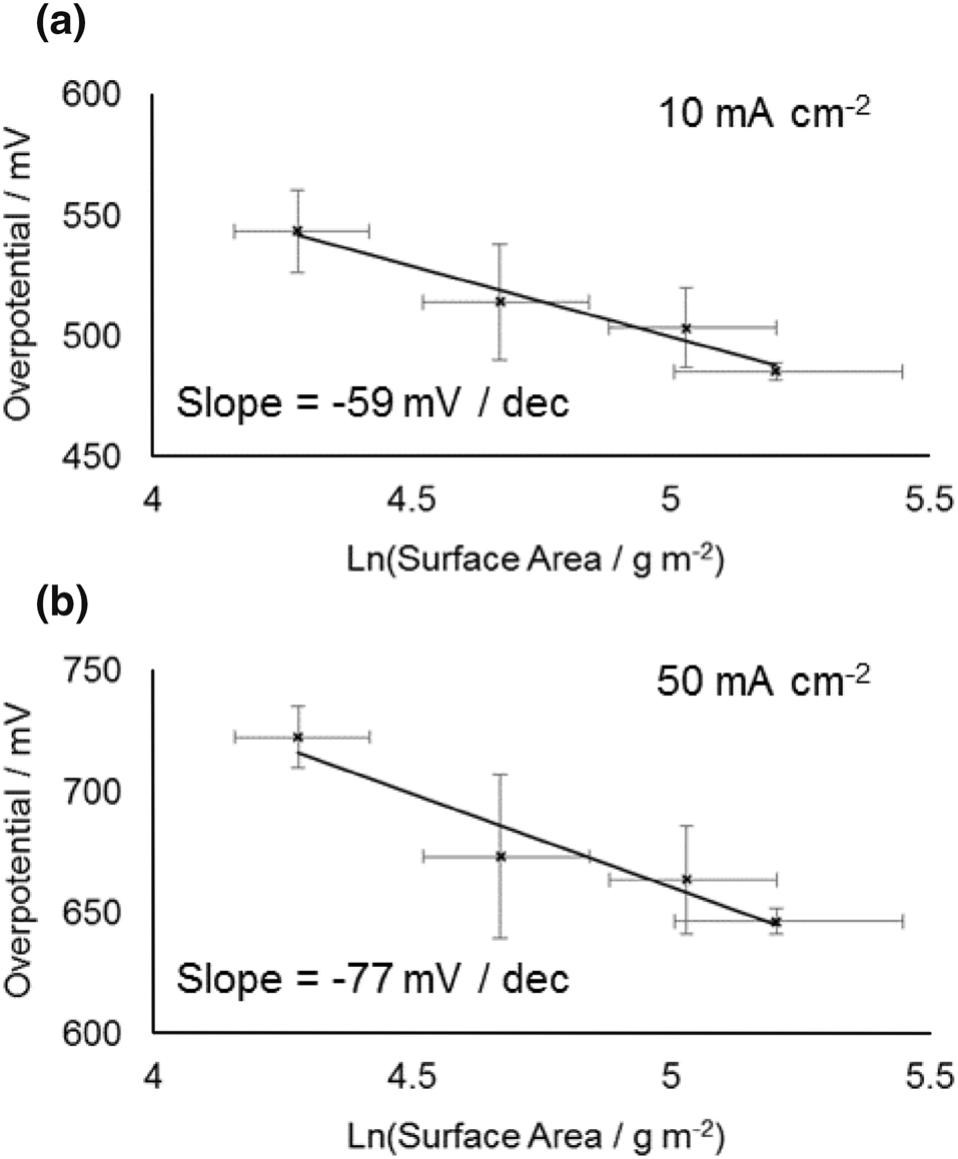
Tafel plot of overpotential against ln(surface area per gram) based on the nanoparticle’s geometric surface area (assuming the nanoparticles to be roughly spherical). The error bars in the y-axis show standard deviation in the mean based on repeat measurements of each point, the error bars in the x-direction show error in the ln(surface area) based on propagation of the standard deviation of the measured particle size distribution

**Fig. 5:**
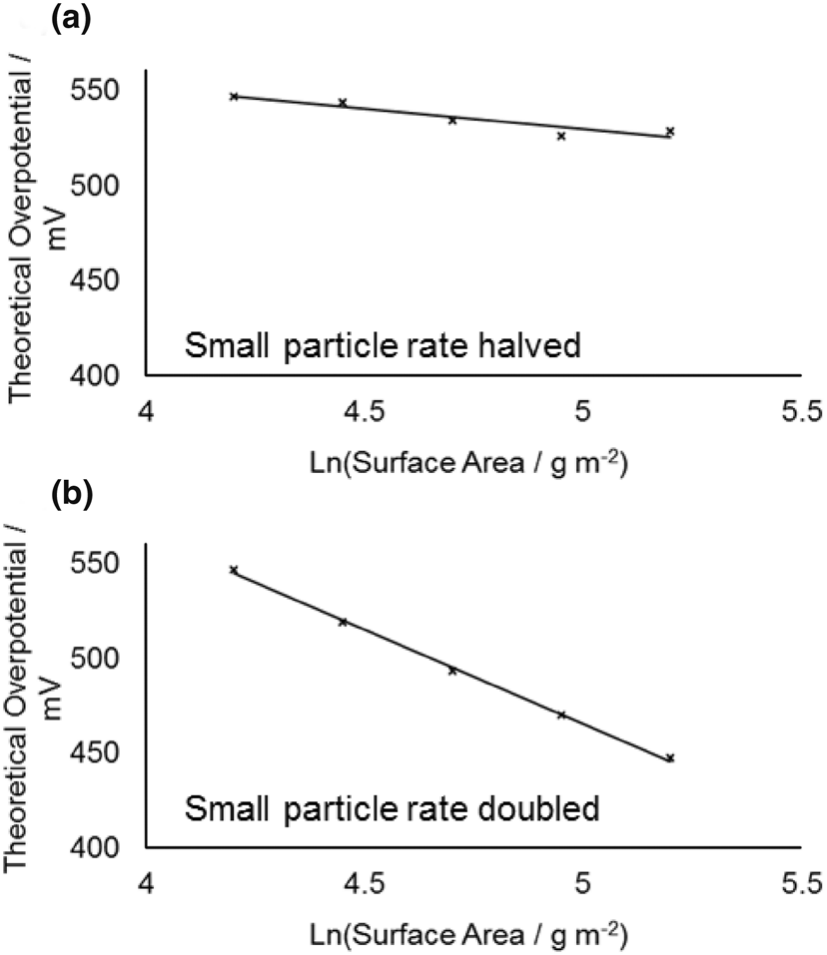
Schematic showing the effect of recalculating the Tafel plot for 10 mA cm^−2^ current density based on the Tafel slope in Fig. 4, but assuming the smallest particles are deemed to have half (**a**) or double (**b**) the rate per surface site of the largest ones and vary linearly for intermediate sizes (details of calculation given in the Electronic Supplementary Material)

These four nanoparticle samples (based on the concentration of cobalt determined by ICP OES) were initially used to prepare working electrodes with a fixed cobalt concentration of 20 µg cm^−2^ by deposition on a glassy carbon electrode. This concentration was chosen to allow us to obtain a baseline activity, where most of the cobalt was accessible for reactants and products to absorb and desorb. This is confirmed by the overpotential required to obtain a current of 10 mA cm^−2^ decreasing from 485 to 454 mV between cobalt electrode loadings of 20 and 45 µg cm^−2^ (for the same sample), showing the active surface area to still be increasing substantially, whereas on increasing to higher loading the decrease in overpotential becomes much less (see Electronic Supplementary Material). Figure 3 shows the average overpotential required to obtain specified current densities of 10 and 50 mA cm^−2^ as a function of mean nanoparticle diameter (the average is based on repeats of the entire process including loading the electrode and catalyst testing). While the error bars are significant a clear trend of overpotential required decreasing with decreasing particle size is seen. This is consistent with the previous observation by Esswein et al. for larger cobalt particles, where it was found this change in overpotential was simply a result of the increased surface area [21]. In their work the linear nature of overpotential versus ln(surface area) plots based on the Tafel equation was used to confirm the decrease in overpotential for smaller particles was purely due to the increased surface area. In the present work the same approach was followed and the Tafel plots for the overpotential required to reach current densities of 10 and 50 mA cm^−2^ are shown in Fig. 4. The surface areas were estimated as the geometric surface area of the nanoparticles, which has been shown previously to be in reasonable agreement with other methods of surface area estimation [29]. It should be noted that due to the semi-logarithmic plot if a fraction of each nanoparticle is inaccessible because of deposition on the surface or contact with other nanoparticles, so long as that fraction is assumed to be constant for different sizes nanoparticles the Tafel slope will not vary. As can be seen, the Tafel slope obtained (59 ± 8 mV dec^−1^) is broadly similar to that reported when varying larger sized Co_3_O_4_ starting particles (47 ± 7 mV dec^−1^) [21]. However, consideration of the errors in measurement, even based on repetition of each point, makes asserting no-deviation from a linear relationship dubious. This can be seen more clearly in a simple thought experiment in which the smallest particles are deemed to have half or double the rate per surface site of the largest ones and vary linearly for intermediate sizes (Fig. 5, assumes the above Tafel slope to be otherwise correct—details of calculation are in the Electronic Supplementary Material). Clearly although the Tafel slope obtained is very different, the plots remain very linear in appearance. It is noteworthy that it has previously been suggested that the Tafel relation could be used to calculate a turnover frequency at zero overpotential as a measure of intrinsic activity for electro catalysed processes [35], but the use of the extrapolation of a ln(TOF) versus overpotential graph makes this method similar reliant on precise knowledge of the TOF (which is typically at best an estimate).

**Fig. 6:**
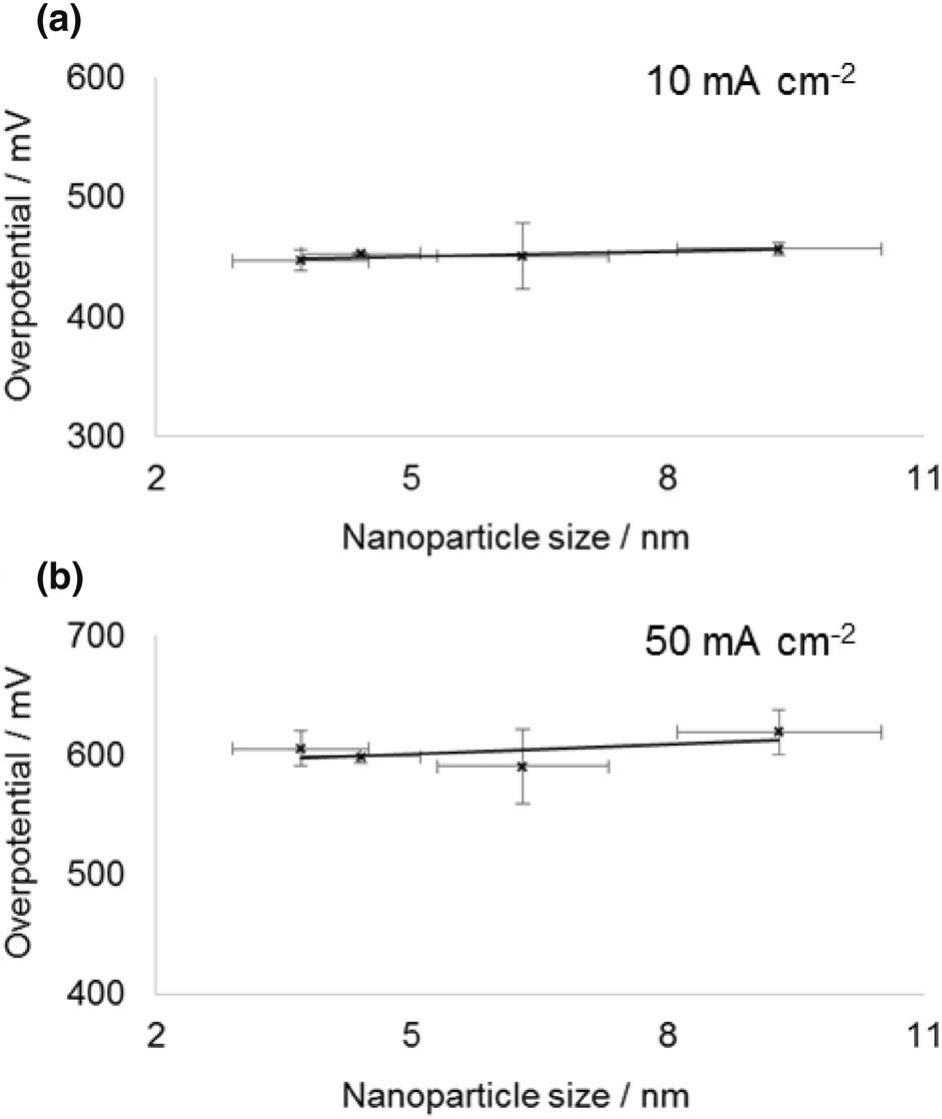
Average overpotential as a function of cobalt nanoparticle size measured at 10 mA cm^−2^ (**a**) and 50 mA cm^−2^ (**b**) using a surface area-normalized electrode loading. The error bars in the y-axis show standard deviation in the mean based on repeat measurements of each point, the error bars in the x-direction show the standard deviation value from the mean of the measured particle size distribution as described above (and not an error in the mean particle size that would be much smaller)

To overcome this problem, the working electrode was instead loaded with a differing amount of cobalt nanoparticles, such that each electrode contained the same geometric surface area of nanoparticles. The average overpotential required to obtain specified current densities of 10 and 50 mA cm^−2^ as a function of mean nanoparticle diameter for a constant surface area can be seen in Fig. 6. This provides definitive evidence that even for small (~ 3.5 nm) particles, which are known to exhibit different oxidation behaviour, there is no significant particle size effect.

**Fig. 7:**
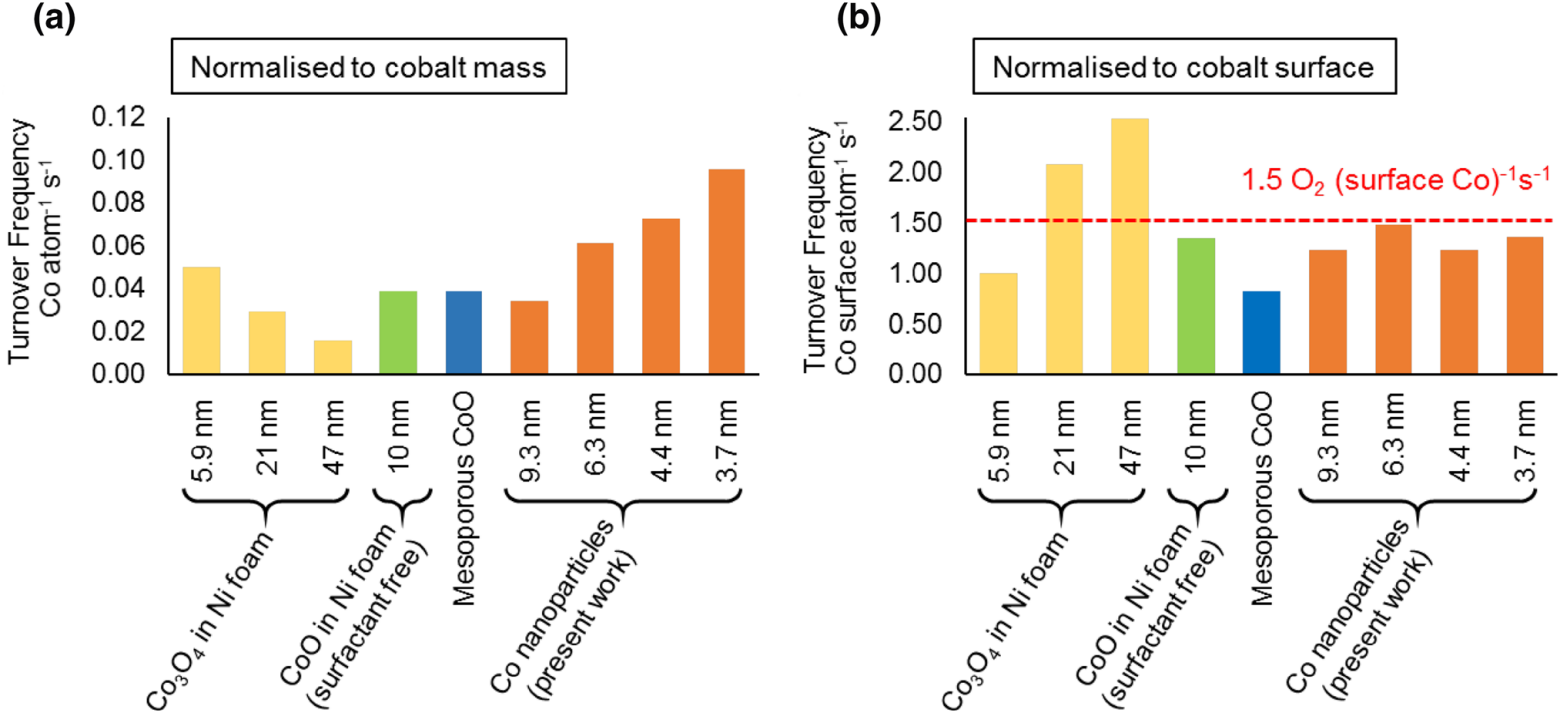
Apparent TOF at η = 500 mV per Co atom (mass activity) (**a**), and per surface Co atom (**b**), for Co_3_O_4_ nanoparticles in Ni foam (surfactant method) [21], CoO nanoparticles in Ni foam (surfactant free) [20], nanocast mesoporous Co_3_O_4_ [24] and the cobalt nanoparticles in the present work. More details of the calculation are given in the Electronic Supplementary Material. All catalysts shown were tested in 1 mol dm^−3^ KOH electrolyte. The geometric area (assuming the particles are spherical) of all nanoparticle materials in their oxide form is used to calculate surface area, assuming (100) surface facets (containing 3.1 × 10^18^ Co atoms m^−2^). For the mesoporous Co_3_O_4_ the reported BET surface area is used as such a calculation is not possible

In addition to investigating whether a particle size effect exists for < 10 nm cobalt nanoparticles, a second aim of the present work was to investigate the intrinsic activity of cobalt nanoparticles—this is important because many previous studies have focussed on practical electrode designs (such as deposition within a conducting foam) [20, 21] or innovative porous cobalt materials [24], but it is not clear how much of the cobalt remains accessible for catalysis. In the present case the cobalt nanoparticles have instead been loaded directly on a flat working electrode surface and with a concentration at which most of the surface area of the nanoparticles is accessible (at most a few layers deep allowing diffusion between them). Knowledge of the intrinsic turnover frequency then allows for the efficiency of 3D electrode designs to be improved to eliminate diffusion restrictions and maximise the useful catalyst area. It is well known that comparison of electrocatalytic activities for different preparations and testing conditions is difficult [32], however it has previously been suggested some comparisons can be drawn for the TOF at a fixed overpotential [36]. In many systems this is difficult to do, because of the uncertainty in the number of accessible surface sites, as discussed in a study of electrodeposited Fe–Co films, where an electrochemical method based on the Co^2+^/Co^3+^ wave during a cyclic voltogram is used to estimate the number of accessible cobalt sites [37].^1^ Figure 7 attempts to compare estimates of the apparent TOF per Co atom and per Co surface atom at a fixed overpotential of 500 mV (for convenient comparison) for the present study and several similar literature studies using nanostructured cobalt. Unsurprisingly, it can be seen that normalising by surface area (Fig. 7b) substantially improves the commonality between the reported results. It can also be seen that for all the nanoparticulate structures the TOF is of a ball park figure a little under 1.5 O_2_ molecule per surface Co site per second. Notably the largest nanoparticle structures reported by Esswein et al. have a slightly larger TOF [21], but this could be the result of either a smaller fraction of the surface being in contact with the support or as result of surface roughening under reaction conditions (the calculation used assumed they are spherical and all of the mean size reported). It can also be seen the nanocast mesoporous Co_3_O_4_ structures have a slightly lower values, suggesting some inaccessibility of some surface sites. Nevertheless this analysis allows us to confirm a “typical” intrinsic TOF for cobalt catalysed OER under these typical conditions.

## Conclusions

In summary, we have shown that small cobalt nanoparticles (4–10 nm) can be used in the OER and under typical alkaline conditions they do not exhibit any particle size effect, in spite of the known differences in oxidation behaviour for lower oxidation state redox couples across this size range. Two methods were used to evaluate this, firstly the standard electrochemical method based on the linearity of Tafel plots and secondly a direct method employing a surface-area normalised cobalt loading on the working electrode. Finally, the use in this study of small quantities of cobalt nanoparticles on a flat working electrode to maximise accessibility, and comparison to several literature studies using cobalt nanostructures, has allowed identification of an estimated intrinsic turnover rate for cobalt surface sites in Co_3_O_4_ catalysed OER under typical alkaline reaction conditions (ambient temperature, pH 14) of order 1 O_2_ molecule per surface Co site per second (η = 500 mV).

## Electronic supplementary material

Below is the link to the electronic supplementary material.

Supplementary material 1 (PDF 294 KB)

## Data Availability

The datasets acquired during and/or analysed during the current study are available from the corresponding author on reasonable request.

## Footnotes

1 It should be noted that the TOFs calculated in this study were at an overpotential of 350 mV at which Co is not active, so no direct comparison can be made.

## References

[1] Lewis NS, Nocera DG (2006). Powering the planet: chemical challenges in solar energy utilization. Proc Natl Acad Sci USA.

[2] Gray HB (2009). Powering the planet with solar fuel. Nat Chem.

[3] Roger I, Shipman MA, Symes MD (2017). Earth-abundant catalysts for electrochemical and photoelectrochemical water splitting. Nat Rev Chem.

[4] Trotochaud L, Boettcher SW (2014). Precise oxygen evolution catalysts: status and opportunities. Scr Mater.

[5] Cheng Y, Jiang SP (2015). Advances in electrocatalysts for oxygen evolution reaction of water electrolysis-from metal oxides to carbon nanotubes. Prog Nat Sci: Mater Int.

[6] Trotochaud L, Ranney JK, Williams KN, Boettcher SW (2012). Solution-cast metal oxide thin film electrocatalysts for oxygen evolution. J Am Chem Soc.

[7] Li X, Walsh FC, Pletcher D (2011). Nickel based electrocatalysts for oxygen evolution in high current density, alkaline water electrolysers. Phys Chem Chem Phys.

[8] Louie MW, Bell AT (2013). An investigation of thin-film Ni–Fe oxide catalysts for the electrochemical evolution of oxygen. J Am Chem Soc.

[9] Deng X, Tüysüz H (2014). Cobalt-oxide-based materials as water oxidation catalyst: recent progress and challenges. ACS Catal.

[10] Khan SA, Khan SB, Asiri AM (2016). Electro-catalyst based on cerium doped cobalt oxide for oxygen evolution reaction in electrochemical water splitting. J Mater Sci: Mater Electron.

[11] Jiao F, Frei H (2009). Nanostructured cobalt oxide clusters in mesoporous silica as efficient oxygen-evolving catalysts. Angew Chem Int Ed.

[12] Reece SY, Hamel JA, Sung K, Jarvi TD, Esswein AJ, Pijpers JJH, Nocera DG (2011). Wireless solar water splitting using silicon-based semiconductors and earth-abundant catalysts. Science.

[13] Zhong DK, Gamelin DR (2010). Photoelectrochemical water oxidation by cobalt catalyst (“Co–Pi”)/α-Fe_2_O_3_ composite photoanodes: oxygen evolution and resolution of a kinetic bottleneck. J Am Chem Soc.

[14] Cobo S, Heidkamp J, Jacques P-A, Fize J, Fourmond V, Guetaz L, Jousselme B, Ivanova V, Dau H, Palacin S, Fontecave M, Artero V (2012). A Janus cobalt-based catalytic material for electro-splitting of water. Nat Mater.

[15] Costentin C, Porter TR, Saveant J-M (2016). Conduction and reactivity in heterogeneous-molecular catalysis: new insights in water oxidation catalysis by phosphate cobalt oxide films. J Am Chem Soc.

[16] Seitz LC, Dickens CF, Nishio K, Hikita Y, Montoya J, Doyle A, Kirk C, Vojvodic A, Hwang HY, Norskov JK, Jaramillo TF (2016). A highly active and stable IrO_x_SrIrO_3_ catalyst for the oxygen evolution reaction. Science.

[17] I.M.M.A. Investment, Commodity and Metal Prices. http://www.infomine.com/investment/metal-pries/. Accessed 05 May 2017

[18] Artero V, Fontecave M, Cobo S, Jacques PA, Dau H, Heidkamp J (2013) Method for preparing a catalyst mediating H2 evolution, said catalyst and uses thereof. US20150090604 A1

[19] Surendranath Y, Kanan MW, Nocera DG (2010). Mechanistic studies of the oxygen evolution reaction by a cobalt-phosphate catalyst at neutral pH. J Am Chem Soc.

[20] Chou NH, Ross PN, Bell AT, Tilley TD (2011). Comparison of cobalt-based nanoparticles as electrocatalysts for water oxidation. ChemSusChem.

[21] Esswein AJ, McMurdo MJ, Ross PN, Bell AT, Tilley TD (2009). Size-dependent activity of Co_3_O_4_ nanoparticle anodes for alkaline water electrolysis. J Phys Chem C.

[22] Wu J, Xue Y, Yan X, Yan W, Cheng Q, Xie Y (2012). Co_3_O_4_ nanocrystals on single-walled carbon nanotubes as a highly efficient oxygen-evolving catalyst. Nano Res.

[23] Liang Y, Li Y, Wang H, Zhou J, Wang J, Regier T, Dai H (2011). Co_3_O_4_ nanocrystals on graphene as a synergistic catalyst for oxygen reduction reaction. Nat Mater.

[24] Tüysüz H, Hwang YJ, Khan SB, Asiri AM, Yang P (2013). Mesoporous Co_3_O_4_ as an electrocatalyst for water oxidation. Nano Res.

[25] Mattioli G, Giannozzi P, Amore Bonapasta A, Guidoni L (2013). Reaction pathways for oxygen evolution promoted by cobalt catalyst. J Am Chem Soc.

[26] McAlpin JG, Surendranath Y, Dincǎ M, Stich TA, Stoian SA, Casey WH, Nocera DG, Britt RD (2010). EPR evidence for Co(IV) species produced during water oxidation at neutral pH. J Am Chem Soc.

[27] Grass ME, Zhang Y, Butcher DR, Park JY, Li Y, Bluhm H, Bratlie KM, Zhang T, Somorjai GA (2008). A reactive oxide overlayer on rhodium nanoparticles during CO oxidation and its size dependence studied by in situ ambient-pressure X-ray photoelectron spectroscopy. Angew Chem Int Ed.

[28] Karim W, Kleibert A, Hartfelder U, Balan A, Gobrecht J, van Bokhoven JA, Ekinci Y (2016). Size-dependent redox behavior of iron observed by in-situ single nanoparticle spectro-microscopy on well-defined model systems. Sci Rep.

[29] Bezemer GL, Bitter JH, Kuipers HPCE, Oosterbeek H, Holewijn JE, Xu X, Kapteijn F, van Dillen AJ, de Jong KP (2006). Cobalt particle size effects in the Fischer–Tropsch reaction studied with carbon nanofiber supported catalysts. J Am Chem Soc.

[30] Iablokov V, Beaumont SK, Alayoglu S, Pushkarev VV, Specht C, Gao JH, Alivisatos AP, Kruse N, Somorjai GA (2012). Size-controlled model Co nanoparticle catalysts for CO_2_ hydrogenation: synthesis, characterization, and catalytic reactions. Nano Lett.

[31] Tsakoumis NE, Walmsley JC, Rønning M, van Beek W, Rytter E, Holmen A (2017). Evaluation of reoxidation thresholds for γ-Al_2_O_3_-supported cobalt catalysts under Fischer–Tropsch synthesis conditions. J Am Chem Soc.

[32] McCrory CCL, Jung S, Peters JC, Jaramillo TF (2013). Benchmarking heterogeneous electrocatalysts for the oxygen evolution reaction. J Am Chem Soc.

[33] Kuo C-H, Mosa IM, Poyraz AS, Biswas S, El-Sawy AM, Song W, Luo Z, Chen S-Y, Rusling JF, He J, Suib SL (2015). Robust mesoporous manganese oxide catalysts for water oxidation. ACS Catal.

[34] Lide DR (2005). CRC handbook of chemistry and physics.

[35] Costentin C, Drouet S, Robert M, Savéant J-M (2012). Turnover numbers, turnover frequencies, and overpotential in molecular catalysis of electrochemical reactions. Cyclic voltammetry and preparative-scale electrolysis. J Am Chem Soc.

[36] Gao M, Sheng W, Zhuang Z, Fang Q, Gu S, Jiang J, Yan Y (2014). Efficient water oxidation using nanostructured α-nickel-hydroxide as an electrocatalyst. J Am Chem Soc.

[37] Burke MS, Kast MG, Trotochaud L, Smith AM, Boettcher SW (2015). Cobalt–iron (oxy)hydroxide oxygen evolution electrocatalysts: the role of structure and composition on activity, stability, and mechanism. J Am Chem Soc.

