# Supplementary material for: Catalysis of the Oxygen Evolution Reaction by 4–10 nm Cobalt Nanoparticles
Source: Top Catal. 2018 Apr 9;61(9):977–85. doi: 10.1007/s11244-018-0923-4 (PMC6435211; doi:10.1007/s11244-018-0923-4)
Supplement: Supplementary file 1 — Supplementary material 1 (PDF 294 KB) [file 11244_2018_923_MOESM1_ESM.pdf]

Electronic Supplementary Material

Catalysis of the oxygen evolution reaction by 4-10 nm cobalt nanoparticles

Edward Locke[a], Shan Jiang[a] and Simon K Beaumont[a]*

[a] Department of Chemistry, University of Durham, South Road, Durham, DH1 3LE, UK.

1. Stability experiments using chrono-potentiometry
2. Overpotential as a function of nanoparticle loading on the electrode
3. Details of modelled Tafel plot for particle size dependent activity
4. Table of TOF calculations
5. Table showing sensitivity analysis for TOF calculations

1. Stability experiments using chrono-potentiometry

Figure S1 shows a stability test for the 3.7 nm particles (selected as most active) measuring the potential required to maintain a constant current density of 10 mA cm^{-2}. This was done with and without a Nafion stabiliser and also compared to the clean glassy carbon reference electrode. In general a stable or only slightly decreasing activity (increasing overpotential) was seen for the sample run in the absence of Nafion, confirming the stability of the catalysts on the timescale of the much shorter CV experiments being analysed in this work. However for both catalyst samples something occurs at 7-8 hours that terminates the reaction – it is not clear if this is mechanical or result of gradual dissolution, so care has been taken to replace the electrolyte and thoroughly wash glassware in between each cyclic voltammetry run.

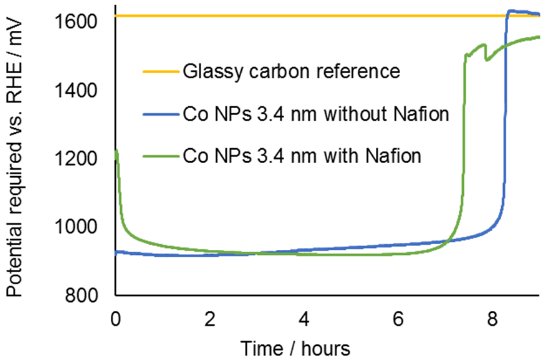

Fig. S1 *Potential required to maintain a current density of 10 mA cm^{-2} as a function of time for a choro-potentiometry experiment using the 3.7 nm nanoparticles with a cobalt loading of 20 µg cm^{-2}*

The second notable feature in Figure S1 is attempts to use Nafion immobiliser to stabilise the particles was unsuccessful, but additionally in the early part of the stability study (up to two hours) it appeared to inhibit catalysis. This is consistent with the observation in CV experiments that Nafion stabilised catalyst was much less active (Figure S2).

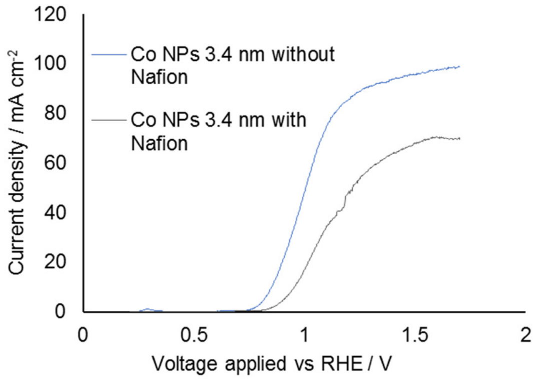

Fig. S2 *Cyclic voltogram sweep from 0 to 1.6 V showing the marked difference in activity with and without Nafion stabilizer for the 3.7 nm nanoparticles.*

2. Overpotential as a function of nanoparticle loading on the electrode.

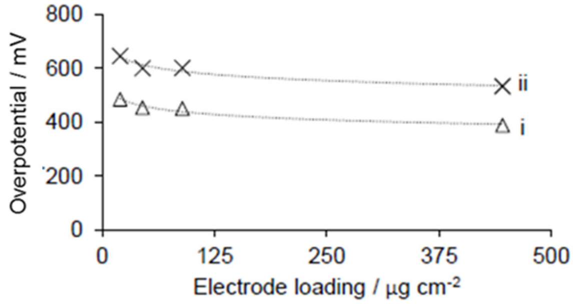

Fig. S3 *Overpotential for (i) 10 and (ii) 50 mA cm^{-2} current densities as a function of the electrode loading for the 3.7 nm nanoparticle catalyst, showing a sharp decrease in the region used for the remainder of the study (near 20 µg cm^{-2}) indicating an absence of significant mass transport limitations are occurring in this loading range*

3. Details of modelled Tafel plot for particle size dependent activity

In order to assess the impact of increasing or decreasing the activity by a factor of two on the Tafel plots the equation of the actual Tafel slope (Main Paper Figure 4) was used to calculate the theorectical overpotential at a number of evenly spaced values of Ln(Surface Area). The surface area was then calculated, and multiplied by an adjustment factor to give an effective surface area either double or half for the smallest size particles and in proportion for the values inbetween to no adjustment for the largest particles. The Tafel slope equation was then used to calculated the overpotential required for these effective surface areas and that adjusted overpotential plotted against the original Ln(Surface Area) values. The results are shown in Main Paper Figure 5, and the calculated values tabulated below.

Doubling the activity per Co surface site for the smallest particles:

Ln(Surface Area / g m^{-2})	Tafel Eqn. Overpotential / mV	Adjustment factor	Original surface area / g m^{-2}	Adjusted surface area / g m^{-2}	Adjusted Overpotential from Tafel Equation / mV
4.20	547	1.00	67	67	547
4.45	532	1.25	86	107	519
4.70	517	1.50	110	165	494
4.95	503	1.75	141	247	470
5.20	488	2.00	181	363	447

Halving the activity per Co surface site for the smallest particles:

Ln(Surface Area / g m^{-2})	Tafel Eqn. Overpotential / mV	Adjustment factor	Original surface area / g m^{-2}	Adjusted surface area / g m^{-2}	Adjusted Overpotential from Tafel Equation / mV
4.20	547	1.00	67	67	547
4.45	532	0.83	86	71	543
4.70	517	0.75	110	82	534
4.95	503	0.68	141	95	526
5.20	488	0.50	181	91	529

4. Table of TOF calculations

Sample	5.9 nm Co_3O_4 in Ni foam	21 nm Co_3O_4 in Ni foam	47 nm Co_3O_4 in Ni foam	10 nm CoO in Ni foam[a]	Meso-porous Co_3O_4[b]	9.3 nm Co	6.3 nm Co	4.4 nm Co	3.7 nm Co
Reference	[1]			[2]	[3]	This work			
Corrected diameter[c] / nm	5.9	21.0	47.0	10.2	NA	10.5	7.1	5.0	4.2
Oxide Surface area[d] /m^2 g^{-1}	167	47	21	96	156	94	138	198	235
Co_3O_4 loading /mg cm^{-2}	1	1	1	1	0.13				
Cobalt loading /mg cm^{-2}	0.580	0.580	0.580	0.648	0.075	0.020	0.020	0.020	0.020
Current density[e] / mA cm^{-2}	190	110	60	165	19	4.5	8.0	9.5	12.5
O_2 formed /10^{-8} mol $cm^{-2}s^{-1}$	49.3	28.5	15.6	42.8	4.93	1.17	2.08	2.46	3.24
Co atoms /10^{-7} mol cm^{-2}	98.5	98.5	98.5	110	12.8	3.40	3.40	3.40	3.40
Surface Co atoms / 10^{-8} mol cm^{-2}	49.0	13.8	6.2	31.7	5.97	0.95	1.40	2.01	2.39
TOF (all cobalt) / s^{-1}	0.05	0.03	0.02	0.04	0.04	0.03	0.06	0.07	0.10
TOF (surface cobalt) / s^{-1}	1.00	2.07	2.52	1.35	0.82	1.23	1.48	1.23	1.36

[a]Synthesized *via* a surfactant free method. [b]Synthesized by a nanocasting method, sample annealed at 35 °C to maximise surface area, supported on electrode using Nafion. [c]Corrected radius based on the assumption all Co of CoO converted to Co_3O_4.[d]Surface areas calculated geometrically assuming spherical particles, except for mesoporous Co_3O_4 for which the reported BET surface area was used. [e]Value reported at 500 mV overpotential, all samples recorder in pH 14.0, 1 mol dm^{-3} KOH.

5. Table showing sensitivity analysis for TOF calculations, taking the example of 9.3 nm Co particles from this work (section 4) and repeating with different shapes or surface terminations.

Assumed shape	Spherical (Diameter = 9.3 nm)	Cubic (Apparent Diameter[a] = 9.3 nm)	Spherical (Diameter = 9.3 nm)	Spherical (Diameter = 9.3 nm)
Assumed surface termination	(100)	(100)	(111)	(110)
Co surface atom density[b] / 10^{18} m^{-2}	3.06	3.06	3.53[c]	8.65
Corrected diameter[d] / nm	10.5	10.5	10.5	10.5
Surface Area of one nanoparticle / nm^2	$\pi(10.5^2) = 346$	$6\times(8.6^2) = 444$	346	346
Volume of NP/ nm^3	$(\pi/6)\times(10.5^3) = 606$	$(8.6^3) = 636$	606	606
Specific Oxide Surface area[e] /m^2 g^{-1}	$((346/606)/6.11)\times10^3 = 93.4$	$((444/636)/6.11)\times10^3 = 114$	93.4	93.4
Cobalt loading /10^{-6} gcm^{-2}	20	20	20	20
Current density[f] / mA cm^{-2}	4.5	4.5	4.5	4.5
O_2 formed /10^{-8} mol $cm^{-2}s^{-1}$	1.17	1.17	1.17	1.17
Co atoms /10^{-7} mol cm^{-2}	3.40	3.40	3.40	3.40
Surface atom density / 10^{-6} mol m^{-2}	5.08	5.08	5.86	14.4
Surface Co atoms / 10^{-9} mol cm^{-2} (of electrode)	$20\times93.4\times5.08\times10^{-12} = 9.51$	11.6	11.0	26.9
TOF (all cobalt) / s^{-1}	0.34	0.34	0.34	0.34
TOF (surface cobalt) / s^{-1}	1.23	1.01	1.07	0.43[g]

[a]Apparent diameter is taken to be the mean of the body diagonal of a cube (max) and edge length of the cube (min) dimensions, for 10.5 nm this requires a cube edge length 8.6 nm. [b] The cobalt surface densities for (100), (110) and (111) were calculated based on the reported crystal structure of Co_3O_4 spinel [4] [c]For the (111) plane the closest plane of Co atoms was used. [d]Corrected radius based on the assumption all Co of CoO converted to Co_3O_4. [e]Surface areas calculated geometrically assuming spherical particles, except for mesoporous Co_3O_4 for which the reported BET surface area was used. [f]Value at 500 mV overpotential, recorded in pH 14.0, 1 mol dm^{-3} KOH. [g]Although the value of TOF obtained here is a factor of ~2 higher, the (110) surface is higher energy and so likely only contributes a small fraction of the surface area in a non-faceted particle.

Electronic Supplementary Material References

1. Esswein AJ, McMurdo MJ, Ross PN, Bell AT, Tilley TD (2009) Size-Dependent Activity of Co_3O_4 Nanoparticle Anodes for Alkaline Water Electrolysis. J Phys Chem C 113:15068-15072.
2. Chou NH, Ross PN, Bell AT, Tilley TD (2011) Comparison of Cobalt-based Nanoparticles as Electrocatalysts for Water Oxidation. ChemSusChem 4:1566-1569.
3. Tüysüz H, Hwang YJ, Khan SB, Asiri AM, Yang P (2013) Mesoporous Co_3O_4 as an electrocatalyst for water oxidation. Nano Res 6:47-54.
4. Will G, Masciocchi N, Parrish W, Hart M (1987) Refinement of simple crystal structures from synchrotron radiation powder diffraction data. J Appl Crystallogr 20:394-401.
